# Prediction of sgRNA Off-Target Activity in CRISPR/Cas9 Gene Editing Using Graph Convolution Network

**DOI:** 10.3390/e23050608

**Published:** 2021-05-14

**Authors:** Prasoon Kumar Vinodkumar, Cagri Ozcinar, Gholamreza Anbarjafari

**Affiliations:** 1 iCV Lab, Institute of Technology, University of Tartu, 51009 Tartu, Estonia; prasoon.vinodkumar@ut.ee (P.K.V.); chagri.ozchinar@ut.ee (C.O.); 2 PwC Advisory Finland, 00180 Helsinki, Finland

**Keywords:** gene editing, deep learning, graph convolutional network, sgRNA, link prediction

## Abstract

CRISPR/Cas9 is a powerful genome-editing technology that has been widely applied in targeted gene repair and gene expression regulation. One of the main challenges for the CRISPR/Cas9 system is the occurrence of unexpected cleavage at some sites (off-targets) and predicting them is necessary due to its relevance in gene editing research. Very few deep learning models have been developed so far to predict the off-target propensity of single guide RNA (sgRNA) at specific DNA fragments by using artificial feature extract operations and machine learning techniques; however, this is a convoluted process that is difficult to understand and implement for researchers. In this research work, we introduce a novel graph-based approach to predict off-target efficacy of sgRNA in the CRISPR/Cas9 system that is easy to understand and replicate for researchers. This is achieved by creating a graph with sequences as nodes and by using a link prediction method to predict the presence of links between sgRNA and off-target inducing target DNA sequences. Features for the sequences are extracted from within the sequences. We used HEK293 and K562 t datasets in our experiments. GCN predicted the off-target gene knockouts (using link prediction) by predicting the links between sgRNA and off-target sequences with an auROC value of 0.987.

## 1. Introduction

Genome engineering is the ability to engineer biological systems that allows the modification of genome and transcription products on target sites. The Clustered Regularly Interspaced Short Palindromic Repeats (CRISPR) and CRISPR-associated Protein 9 (Cas9) [1,2,3,4] is one of the most widely used genome-editing technologies due to its pellucid mechanism, simple operation and higher degree of flexibility and accuracy in cutting and pasting genes. CRISPR/Cas9 system, originally derived from the immune defense mechanism of bacteria and archaea, requires three important components in the process of gene editing: Cas9 protein, a chimeric single-guide RNA (sgRNA) and PAM motif (protospacer adjacent motif) [5]. NGG is the most commonly used PAM type, where N represents any base of A, C, T, G [6,7,8]. The targeting efficiency and specificity of the CRISPR/Cas9 system depends on the following fundamental requirements [9,10]:

1. sgRNA (sequence of 20 nucleotides in length) needs to be complementary with its targeting genome sequence.
2. PAM (three nucleotide motifs on the target sequence and a prerequisite for Cas9 protein cleavage) needs to be located around the target site [9,11,12].
3. Cas9 protein cleaves the target DNA at the site, three bases upstream of PAM, under the guidance of sgRNA sequence.

During CRISPR/Cas9 gene editing, sgRNA can influence other regions, resulting in unintended cleavage of DNA sequence, referred to as “Off-targets” [8,13,14,15]. Off-target mutations could lead to major problems when applying CRISPR/Cas9 gene editing to clinical applications and the focus of study on CRISPR/Cas9 is to reduce these off-target mutations. For this reason, predictive models are required to accurately predict the off-target mutations in CRISPR/Cas9 gene editing.

Many off-targets detecting methods such as GUIDE-Seq [16,17,18] method, HTGTS (High Throughput Genome-wide Translocation Sequencing) method [19], BLESS (direct in situ breaks labeling sequencing) method [20], Digenome-seq [21,22,23] method and IDLV (Integration-Deficient Lentiviral Vector capture) method [24,25] were developed to predict the off-target mutations in CRISPR/Cas9. However, these methods could not detect all off-target sites of a specific sgRNA and had a low detection accuracy. Other off-target prediction methods such as CFD (Cutting Frequency Determination) [26], CROP-IT score method [27], CCTop score [28] and MIT score [8] just calculated mismatch scores and were vulnerable to experimental variation. Moreover, these methods did not consider the growing CRISPR/Cas9 off-target data for continuous self-learning. Learning-based prediction models are required to effectively predict off-target mutations in a whole genome as the cutting efficacy of sgRNA varies significantly [9,29,30].

Machine learning has been gradually applied to sgRNA activity prediction [6] and off-target site prediction [6,31]. However, these traditional machine learning methods cannot take raw features from large, annotated datasets and use them to identify the patterns buried inside them. Deep learning algorithms is a powerful approach for learning complex patterns and has led to multiple performance breakthroughs in many research fields, including computer vision [32,33,34,35,36,37] and natural language processing [38,39,40]. However, very few prediction models have implemented the concept of deep learning into the sgRNA off-target propensity prediction problem.

*DeepCRISPR* [41], *CNN_Std* [42], *AttnToMismatch_CNN* [43] and *CnnCrispr* [44] have used convolutional neural network (CNN) to predict sgRNA off-target activity by implementing automatic recognition of sequence features but implemented a complex process of feature extraction and off-target prediction that is difficult to be understood and replicated by researchers. *DeepCRISPR* extracts epigenetic features of DNA limiting its application to selective cell types and is not user friendly [45]. *CNN_Std* attempted to downsize the fully connected layer making it difficult to down-sample 23 × 1 feature maps [46]. In *AttnToMismatch_CNN* model, the authors did not use the PAM region of the sequence for feature extraction, which has a significant role in targeted gene editing by CRISPR/Cas9 system. *CnnCrispr* uses the *GloVe* embedding model, which limits the use of this model by researchers due to its complexity in feature extraction process.

Graphs are sets of data structures that can model a set of objects (as nodes) and their relationships as edges. In many biological networks, graphs can be used to capture interactions between bio-molecules such as RNA, DNA and proteins [47]. Graphs are non-Euclidean data structures and standard neural networks, such as CNN, cannot efficiently handle graph inputs and can cause loss of dependency information of the nodes. Graph neural networks (GNN) are considered more efficient in graph analysis than standard neural networks due to their efficient handling of input and dependency information. Even an untrained GNN with a simple architecture can perform well by identifying hidden patterns that are difficult to be extracted using standard neural networks [48,49]. Efficient pattern recognition algorithms are required for predicting off-target efficacy of sgRNA in the CRISPR/Cas9 system that is easy to implement and can extract features from sequences. We believe that a graph-based deep learning approach could be efficient in identifying complex hidden patterns for off-target prediction in CRISPR/Cas9 gene editing.

The purpose of this research is to introduce a novel graph-based approach to predict off-target efficacy of sgRNA in CRISPR/Cas9 system that is easy to understand and replicate by researchers. This is achieved by using link prediction method, a form of graph analysis, to predict the presence of links/edges between the sgRNA and the off-target inducing target DNA sequences (positive links) and the absence of links between sgRNA and target sequences that did not produce off-target effects (negative links). The positive and negative links are encoded and labelled as 1 and 0, respectively, by using many in-built functions and binary operators provided by StellarGraph [50]. A network graph from the off-target dataset is created by setting the unique sgRNA and target DNA sequences as nodes and by creating links between sgRNA and off-target producing target sequences.

The main objectives of this research are:

1. to develop a graph-based approach for off-target prediction in the CRISPR/Cas9 system that is easy to understand and implement by researchers,
2. to use a link prediction method and to predict the presence of links between sgRNA and off-target producing target sequences,
3. to use a graph convolutional network (GCN), a powerful neural network model used for performing representation learning of network graphs created from off-target data set,
4. to provide features for sequences (nodes) in the graph by extracting features from within the sequences,
5. to handle the imbalance in the off-target dataset using cluster data sampling, by random sampling of sequences in every cluster in the network graph, and,
6. to make use of StellarGraph [50] library that enables researchers to easily identify patterns and implement graph machine learning.

## 2. Background

### 2.1. Graph Convolutional Network

Graph convolutional network (GCN) [51] is a powerful neural network architecture for deep learning on graph *G*,

(1) G=(V,E)

where *V* is the vertices/nodes and *E* is the edges/links in a graph.

GCN takes the matrix representation of input feature matrix (*X*) and adjacency matrix (*A*) as inputs. Input feature matrix (*X*) is described as,

(2) $$X=N*F^{0}$$

where *N* is the number of nodes and $F^{0}$ is the number of input features for each node.

Adjacency Matrix (*A*) is the matrix representation of the graph described as,

(3) A=N∗N

where *N* is the number of nodes in the graph.

The number of edges connected to the nodes, also known as “node degree”, is calculated and the feature representations are normalized by the computed node degree to avoid vanishing or exploring gradients and to avoid any issues for stochastic gradient algorithms that are sensitive to the scale of each of the input features.

A hidden layer in the GCN can be described as,

(4) $$H^{i}=f\left(H^{i-1},A\right)$$

where $H^{0}$ is the input feature matrix and *f* is the propagation. Each hidden layer *H*, represents the N×F of the feature matrix, with each row being a feature representation of a node. Using the propagation rule *f* at every layer, these features are aggregated to form the next layer’s features to make them increasingly abstract at each consecutive layer.

The adjacency matrix (*A*) is then transformed by adding it with an identity matrix (*I*) to add a self-loop to each node, as the aggregated representation of a node does not include its own features unless the nodes have a self-loop.

(5) $$\hat{A}=A+I$$

Node degree of the transformed adjacency matrix ($\hat{A}$) is calculated and the transformed adjacency matrix is normalized by the computed node degree similar to the feature matrix. The feature matrix and adjacency matrix are normalized to avoid the change of scale of feature vectors when performing the matrix multiplication of the adjacency matrix and the feature matrix. Thus, the propagation rule would look like this,

(6) $$f\left(X,A\right)=z\left(D^{-\frac{1}{2}}\times \hat{A}\times X\right)$$

where z is a non-linear function (ReLu function). Multiplying the normalized feature matrix (*X*) with a diagonal node degree of the transformed adjacency matrix *($D^{-\frac{1}{2}}$)* and ($\hat{A}$) will take the average of neighboring node features.

### 2.2. Link Prediction Using Stellargraph

StellarGraph API provides many in-built classes such as “*EdgeSplitter*” and “*FullBatchLinkGenerator*”, which can be used to work on nodes and links for link prediction. “*EdgeSplitter*” class, provided by StellarGraph, is used to randomly sample the edges by keeping all the sequences in the train and test set, instead of taking a subset of sequences [50].

(7) edge_splitter_test=EdgeSplitter(G)

This will return a train graph (that shows whether a link should exist between two sequences) for training the model and a test graph for evaluating the performance of the model. Both the train graphs and test graphs will have the same number of sequences but the number of links between the sequences will differ as some of the links will be sampled for training and testing the link prediction classifier.

“*FullBatchLinkGenerator*” class, provided by StellarGraph, is used to create link generators for the train and test link examples to the model. The “*flow*” method supplies the links as a list of nodes. The link generators will feed the list of nodes obtained from “flow” method and feed it to the Keras model, along with the corresponding binary labels that indicate the nodes true or false links in the form of features array and sparse adjacency matrix. The “*method*” parameter should be specified to select the right preprocessing algorithm for the adjacency matrix.

(8) $$train\_gen=FullBatchLinkGenerator(G\_train,method=‘‘gcn^{\prime \prime })$$

(9) train_flow=train_gen.flow(edge_ids_train,edge_labels_train)

Final link classification layer takes a pair of node embeddings produced by the GCN model as input and produces corresponding link embeddings by applying a binary operator and passes it through a dense layer.

The input and output tensors of the GCN model for link prediction are exposed using the GCN.in_out_tensors method provided by StellarGraph [50].

(10) x_inp,x_out=gcn.in_out_tensors()

The *x_out* value is a TensorFlow tensor that holds a 16-dimensional vector for the nodes requested when training or predicting. Predictions are reshaped from (X,1) to (X). GCN and prediction layers are stacked into a keras model and the loss is specified [50].

## 3. Related Works

### 3.1. DeepCrispr

#### 3.1.1. Architecture

*DeepCrispr* applies the rules of auto-encoders to predict off-target propensity and target cleavage site of sgRNA by extracting epigenetic and sequence features of DNA using a deep unsupervised learning. A hybrid DL model with pre-trained DCDNN-based network (as a parent network) and CNN, is extended by reusing pre-trained parent network for off-target prediction. Hence, the model consists of two pre-trained DCDNN-based encoders, one merged layer and CNN layers. Using a deep convolutional denoising neural network (DCDNN)-based autoencoder, unlabeled sequences are trained in unsupervised manner to learn an efficient feature representation of the unlabeled data using encoding and decoding, which will be fitted for building the model. Using CNN model efficacy of sgRNA is predicted. The training process learned the weights of CNN network and tuned the weights of parent network, creating two different “baby networks” and their weights are used for predicting off-target efficacy of sgRNA.

#### 3.1.2. Feature Extraction

For feature extraction, 20-base pair (bp) unlabeled sgRNA sequences with NGG PAM extracted from coding and non-coding regions with different epigenetic information curated from 13 human cell types are given as input for the model. sgRNA sequences and their possible off-target loci, treated as sequence pairs, are encoded using two-part encoding. These sequence-pairs are fit into parent network for feature extraction and the outputs of this network are combined and provided as input for CNN.

#### 3.1.3. Performance

The dataset used for this research consists of human sgRNA whole genome off-target data detected by *GUIDE-seq*, *Digenome-seq*, *BLESS*, *HTGTS* and *IDLV*. The dataset consists of 30 sgRNA from two different cell types (*HEK 293* cell line with 18 sgRNA and *K 562 t* cell line with 12 sgRNA), with a maximum of six nucleotide mismatches using *Bowtie*. As the off-target dataset is heavily imbalanced, bootstrapping sampling was done from minor samples to obtain same number of samples as major samples, alleviating the data imbalance. The results of *DeepCrispr* are compared with the results of *CFD*, *MIT*, *CROP-IT* and *CCTop* on this dataset. *DeepCrispr* outperformed all the other models with improved performance to reduce false positives in predicting off-targets.

#### 3.1.4. Review

*DeepCrispr* extracts epigenetic features of DNA to predict off-target propensity and target cleavage site of sgRNA but epigenetic features are highly volatile and have hypothetical dependency on cell state and cell type, which limits its application to selective cell types and cross-species prediction [45]. It is unclear if the epigenetic features will have any specific impact on the model prediction results. *DeepCrispr* uses the largest dataset available to train the model but the article of *DeepCrispr* did not provide detailed information about test data and test results [44]. The number of negative samples is much larger than the number of positive samples in the off-target dataset. The authors of *DeepCrispr* performed multiple experiments but they did not remove common data between training and testing datasets in their first experiment. For other experiments, some of the labeled and unlabeled data were observed to be similar during pre-training of unlabeled data [52]. On comparing the training and test loss curves, over-fitting and under-fitting issues were observed, which leads to poor performance of the model [46].

### 3.2. CNN_Std

#### 3.2.1. Architecture

A deep standard CNN, (*CNN_Std*) [42], uses deep CNN and a deep feedforward neural network (FNN) to predict off-target mutations by constructing a two-dimensional matrix by only using sequence features. The architecture of *CNN_Std* consists of a convolutional layer to extract matching information of sgRNA–DNA sequence pairs, a batch normalization (BN) layer with *ReLu* as the activation function to reduce internal covariate shift and allow higher learning rates, a global max-pooling layer to verify the mismatches modeled by BN layer, two fully connected dense layers with a dropout layer used on the last dense layer to randomly mask portions of the output to avoid over-fitting and a final output layer consisting of two neurons connected to previous layers. The FNN model architecture consists of an input layer, several hidden layers and an output layer with *softmax* as the activation function to convert each neuron output into probability. For both FNN and CNN models, best performance under five-fold stratified cross-validation, *Adam* algorithms (to optimize cross-entropy loss function) and Mini-batch gradient descent (to reduce gradient variance) are adapted.

#### 3.2.2. Feature Extraction

CNN from computer vision is adapted by processing sgRNA-DNA sequence pair with length of 23 (3 bp PAM adjacent to the 20 bases) into a 4 × 23 matrix, using “XOR” coding design, instead of two-dimensional image with color channels. Each base, (Adenine, Cytosine, Guanine and Thymine), in sgRNA and target DNA sequences are encoded as one of the four one-hot vectors [1,0,0,0], [0,1,0,0], [0,0,1,0] and [0,0,0,1]. The mutated information in sgRNA-DNA sequence pair is encoded by deriving a four-length vector by encoding mismatched bases with the OR operator. This encoded code matrix of sgRNA-DNA is used for the CNN-based models and the vectorized form of this matrix is used for the traditional ML models and deep FNN.

#### 3.2.3. Performance

The CRISPOR off-target dataset is used for training, testing and validation, which contains 26,034 presumed off-targets including 143 validated off-targets, having a mismatch count of up to four with one of the PAM such as NAG/NGA/NGG. For additional evaluation, *GUIDE-seq* off-target dataset containing 28 off-targets among 403 potential off-target sites is used, which is excluded from CRISPOR dataset during training. On the CRISPOR dataset, *FNN_3layer* and *CNN_Std* achieved the best performance under stratified five-fold cross-validation and demonstrated progress over traditional ML models such as GBR, random forest (RF) and logistic regression (LR). On the *GUIDE-seq* dataset, *CNN_Std* achieved the highest true positive rate demonstrating the best generalization performance among other prediction models.

#### 3.2.4. Review

*CNN_Std* achieved high accuracy in the CRISPOR dataset by only using sequence features constructing a two-dimensional input matrix using the “XOR” coding design. Similar to *DeepCrispr*, *CNN_Std* had a poor performance due to over-fitting and under-fitting issues. *DeepCas9* [53] and *CNN_Std* have a similar network architecture, using only one multi-scale convolution layer, but the input size of *DeepCas9* is different (30 nucleotides). *CNN_Std* attempted to downsize the fully connected layer by utilizing a maximum pooling layer with a window size 5 × 1 and stride 5 as CNN can abstract features by convolution but it is not possible to perform down-sampling for 23 × 1 feature maps [46].

### 3.3. AttnToMismatch_CNN

#### 3.3.1. Architecture

An attention-based transformer, a deep-learning neural network architecture is used by *AttnToMismatch_CNN* [43] for off-target specificity prediction of the CRISPR/Cas9 system. The architecture of *AttnToMismatch_CNN* consists of embedding layers to encode each position of the sgRNA and DNA sequence pair into a vector representation and encode into a matrix, a transformer layer with encoder and decoder parts to produce output with dimension same as the input, a CNN layer with two *Conv2d* and two *Maxpooling* layers interleaved and a fully connected layer with *softmax* function to predict probability of sgRNA as positive or negative samples. Five-fold cross validation and leave-3-sgRNAs-out scenario were performed to evaluate the model.

#### 3.3.2. Feature Extraction

Base-pairs from each position of aligned sgRNA and DNA sequences are extracted forming 16 different types. Depending on the input sequence length of the dataset, 20 base pairs are extracted from CRISPR/Cas9 dataset. Raw feature importance, the average loss score obtained by calculating eventual losses and mean square losses for regression by perturbing each input feature across all samples is normalized by summing all feature importance values and provided as weights for the model.

#### 3.3.3. Performance

The off-target dataset was created by collecting 656 off-target sites used in *DeepCrispr* model as positive samples and around 165,000 sgRNA-DNA mismatch pairs from *Cas-OFFinder* as negative samples. On comparing the performance of *AttToMismatch_CNN* model with other models such as RF and GBT, *AttToMismatch_CNN* outperformed other models by a margin of 10% when evaluated by AUC-ROC and PR-AUC metrics and around 20% margin in the five-fold cross validation and leave-3-sgRNAs-out scenarios. This model improves true positive rate and reduces the false positive rate with the application of embedding and transformer layer in encoding extracted sequence features into vectors.

#### 3.3.4. Review

The process of encoding of sequence features into vectors in this model is inspired by the word embedding technique in natural language processing (NLP). The off-target dataset used in this study is highly imbalanced and the authors have mentioned that they have over-sampled the positive samples in every mini-batch making it equal to negative samples but did not give detailed information of how they over-sampled the positive samples. Negative samples of the off-target dataset constructed using *CasOFFinder* is very similar to the positive samples used from *DeepCrispr* model. Input perturbation component used for identifying feature importance did not show any difference for features other than first and second positions of 5’ end of the sgRNA. For extracting features from sgRNA-DNA sequence pair, the authors used 20 base-pairs from the sequence-pairs leaving PAM region, which is very crucial for predicting off-targets in CRISPR/Cas9 system.

### 3.4. CnnCrispr

#### 3.4.1. Architecture

*CnnCrispr* [44] predicted the off-target propensity of sgRNA using CNN and biLSTM. The architecture of this model consists of an embedding layer that accepts a two-dimensional vector matrix of *GloVe* model created from a co-occurrence matrix, a biLSTM network with five convolution layers and two full connection layers to extract context features from input, batch normalization and dropout layers to prevent model over-fitting and output layer with *softmax* and *sigmoid* functions as activation functions to obtain results of classification and regression model. The *Adam* algorithm is used to optimize the loss function and initial learning rate is set to 0.01 for training the model.

#### 3.4.2. Feature Extraction

Similar to *AttToMismatch_CNN*, features are extracted by aligning sgRNA-DNA sequences forming 16 different types of base-pairs set with a unique index value and encoded the sgRNA-DNA sequence-pair for *GloVe* embedding. A pre-processed co-occurrence matrix is created from the sequence-pairs and trained using the *GloVe* model to learn word vectors and produce embedded word vector representation of the base-pairs.

#### 3.4.3. Performance

*CnnCrispr* was trained on the off-target dataset of the *DeepCrispr* model (*HEK 293* cell line with 18 sgRNA and *K 562 t* cell line with 12 sgRNA) with 80% of the samples for training and 20% for testing processes. A comparative study is done on the *DeepCrispr* off-target dataset by comparing the performance of *CnnCrispr* with other models such as *CFD*, *MIT* and *CNN_Std*. *CnnCrispr* outperformed all the models by achieving high AUC values of ROC and PRC curves. When comparing to the test set results of *DeepCrispr*, *CnnCrispr* achieved better auPRC values. Similar to the *AttToMismatch_CNN* model, leave-1-sgRNA-out and 29-fold cross-validation scenarios were performed to validate the model’s performance. *CnnCrispr* outperformed all the other models in both the scenarios.

#### 3.4.4. Review

*CnnCrispr* implemented the word embedding technique to encode sequence features in vector model as performed in the *AttToMismatch_CNN* model. The authors have avoided the unknown influence of artificial feature construction on prediction results by using the *GloVe* vector model, which created a co-occurrence matrix for base-pairs by extracting sequence information of sgRNA and corresponding DNA sequences, providing a detailed analysis of position of nucleotides in sgRNA-DNA sequence pairs. The use of the *GloVe* embedding model to extract sequence information is a novel and innovative approach but limits the application of the *CnnCrispr* model by researchers for off-target prediction due to the complexity of the feature extraction process.

## 4. Materials and Methodology

### 4.1. Dataset

The experimental data used in this study are from the attachment provided by the *CnnCrispr* article. This dataset has been used for off-target prediction by *DeepCrispr* and *AttnToMismatch_CNN* models. Data are available from the “off-target data” file and can be downloaded from *CnnCrispr*. Data were obtained by curating the human sgRNA whole-genome off-target profile data detected by GUIDE-seq, Digenome-seq, BLESS, HTGTS and IDLV. This dataset includes 29 unique sgRNAs by concatenating data from two different cell types: HEK293T cell line and its derivatives (18 sgRNAs) and K562 (12 sgRNAs), accounting for a maximum of six nucleotide mismatches. The dataset obtained from *CnnCrispr* model contains the labels of off-target producing sites as “1” and the labels of other sites as “0”. Source links for the dataset can be found in the Data Availability section.

The obtained dataset was validated for null values and the length of sgRNA and target sequences were validated to be of the same length (23 nucleotides in each sequence). A case-sensitive validation is performed on the sequences to verify if the sgRNA and DNA sequences do not contain any characters other than upper-cased bases, *A*, *C*, *G* and *T*, referring to the nucleotides, Adenine (*A*), Cytosine (*C*), Guanine (*G*) and Thymine (*T*), respectively.

### 4.2. Graph

**Figure 1:**
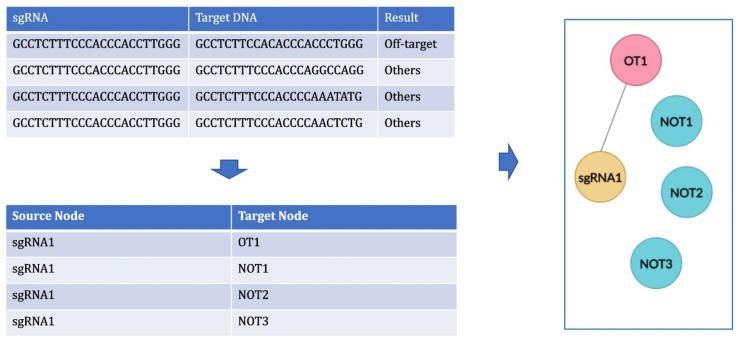
Creating network graph from off-target dataset.

After validating the dataset, we created a network graph using StellarGraph [50] for link prediction using GCN. “Nodes” and “Edges” are required to generate network graph from the off-target dataset. All the unique sequences in the dataset including sgRNA and target DNA sequences are made as nodes in the dataset. An edge will have a start node and a destination node or target node. All the sgRNA sequences were set as start nodes and all target DNA sequences that could induce off-target effects were set as target node for the edges in this graph. The graph contains 29 clusters based on the 29 unique sgRNA sequences forming links with their corresponding sgRNA and off-target sequences. The clusters indicate the sgRNA sequences and its corresponding off-target and other sequences. OT and NOT are the target DNA sequences that are differentiated based on the labels set in the dataset corresponding to the result of off-targets set by the authors of *CnnCrispr*. All the target sequences that produced off-target effects (with label as ‘1’) are named as “OT” and target sequences that did not produce any off-target effects (with label as ‘0’) are named as “NOT” as shown in Figure 1. The naming of the sequences is done as follows:

1. to create the graph with only positive links (links between sgRNA and off-target inducing target DNA (OT) sequences);
2. to create balanced clusters containing an equal number of OT and NOT sequences for every sgRNA cluster using cluster data sampling.

The sequence names, OT and NOT, are discarded during link prediction and not saved as labels for nodes. This is done to make sure that GCN model can accurately predict the presence and absence of links between the sequences by using only the features extracted from the sequences and not based on these sequence names.

**Figure 2:**
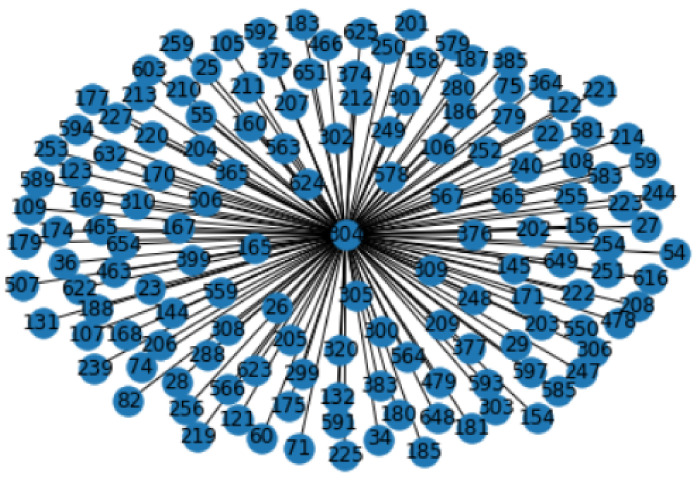
Subgraph of one sgRNA cluster with its corresponding OT sequences.

All the unique sequences in the graphs are numerically encoded forming unique sequence ID for each of the sequences. The sequence ID for these sequences are generated by alphabetically sorting all the sequences, including sgRNA, OT and NOT, in a pandas data frame and then numerically encoding the sequences. A subgraph of 1 sgRNA cluster with its corresponding potential off-targets (OT) created using NetworkX [54] is drawn in Figure 2. The labels of the nodes are the unique sequence IDs generated for the sgRNA and target sequences. The node with the label “804” in the center is the sequence ID for an sgRNA and its neighbor nodes are its corresponding off-target sequences.

### 4.3. Cluster Data Sampling

**Figure 3:**
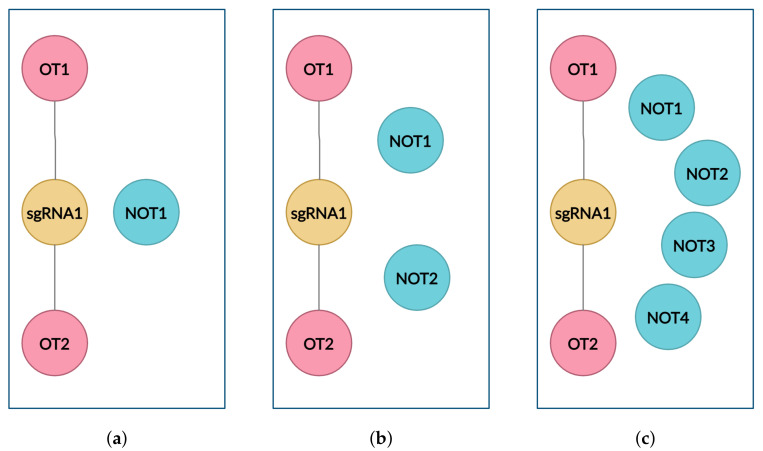
Cluster Data Balancing of OT and NOT sequences in an sgRNA cluster. The imbalanced towards OT scenario, (**a**); the OT and NOT samples are balanced, (**b**); the imbalanced towards NOT scenario, (**c**).

As the dataset is highly imbalanced, the model needs to be trained and tested on a balanced dataset, where the OT and NOT samples are balanced as shown in Figure 3b. Unlike the leave-sgRNA-out scenario that was used in previous models to remove the imbalance between samples, cluster balancing is done to balance the positive and negative samples for each sgRNA cluster. This can be achieved by randomly sampling the NOT sequences with an equal count of OT sequences in an sgRNA cluster, to make sure that all the sgRNA clusters will have equal count of OT and NOT sequences.

The cluster is also sampled in imbalanced scenarios as shown in Figure 3a,c, where the NOT sequences are randomly sampled with respect to OT sequences. For the imbalanced towards NOT scenario (imbalanced_NOT clusters), as shown in Figure 3c, NOT sequences are randomly sampled with twice the amount of OT sequences and for the imbalanced towards OT scenario (imbalanced_OT clusters), as shown in Figure 3a, NOT sequences are randomly sampled with almost half the amount of OT sequences in an sgRNA cluster.

In all scenarios, the count of sgRNA and OT sequences remain unchanged and only the NOT sequences were randomly sampled depending on the count of OT sequences. The total amount of sgRNA, OT and NOT sequences in all 3 scenarios are shown in Table 1. As observed in Table 1, sgRNA and OT sequences remain unchanged for all 3 scenarios and only NOT sequences are randomly sampled depending on the count of OT sequences as mentioned.

### 4.4. Feature Extraction

As all the sequences are numerically encoded, features for these sequences need to be provided, which will enable the GCN model to identify the sequences. The performance of the model was tested by giving two different types of features extracted from within the sequences—position and occurrences of nucleotides in the sequence. These features uniquely identify the sequences in a network graph.

#### 4.4.1. Case Study 1: Nucleotide Occurrence

**Figure 4:**
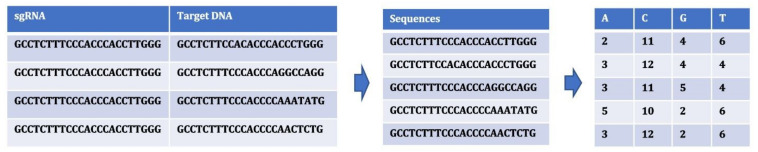
Extracting features for nodes using occurrences of nucleotide in sequences (*k* = 1).

The occurrences of nucleotides in a sequence was extracted. The occurrences of nucleotides can be determined by providing different sizes of *k*-mers. The choice of *k*-mers, substrings of length *k* contained within a biological sequence, has different effects on sequence assembly. We tried to extract features from within the sequences by providing different *k*-mer sizes. *k* values of 1, 2 and 3 were provided to obtain the occurrences. As shown in Figure 4, for k-mer size of 1, the features were A, C, G and T. The number of features for the sequences in this case depend on the *k* values of 1, 2 and 3 as 4, 16 and 64, respectively.

#### 4.4.2. Case Study 2: Nucleotide Position

**Figure 5:**
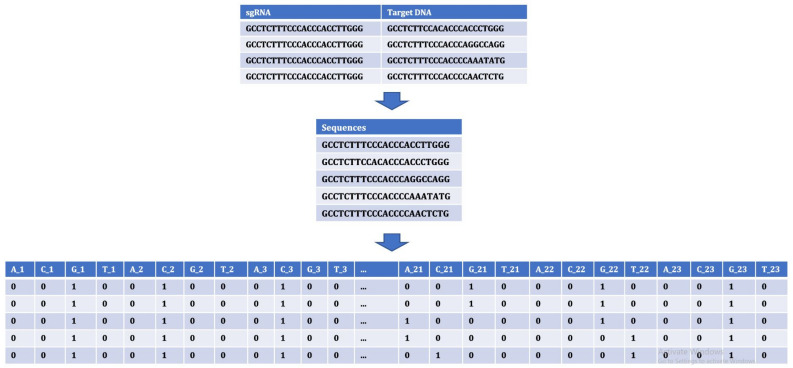
Extracting features for nodes using position of nucleotides in the sequences.

To generate features for nodes by considering the position of the nucleotides, 92 different features were formed based on the possibility of 4 nucleotides occurring at 23 positions in the sequence. Based on the presence and absence of nucleotides in the position, the values were entered as 1 and 0, respectively, as shown in Figure 5.

### 4.5. Graph Analysis

A network graph from the off-target dataset was created by forming nodes and edges. For nodes, a pandas dataframe was created. All the sequences (sgRNA, OT and NOT) were taken as nodes and encoded with sequence IDs. Sequence-based features were extracted from within the sequences and set as column names and column values for the nodes dataframe. For edges, the sgRNA and target DNA sequences that induce off-target (OT) were taken and encoded with sequence IDs in another pandas dataframe. Using StellarGraph, a graph was created by giving these two dataframes as nodes and edges.

Once the graph was created, link prediction was performed to predict whether a link or edge in a graph should exist by performing binary classification using the in-built functions provided by the StellarGraph. The “*EdgeSplitter*” function was used to carefully split the network graph into a training graph, training set, test set and an independent test graph. The training graph was used to compute sequence embeddings. The training set and test set were used to train and test the model on positive and negative edges that were not used for computing sequence embeddings. An independent test graph was created, which contains positive and negative edges not used in computation process, training and test sets, to calculate the Area under the Receiver Operating Characteristic Curves (auROC) values, a performance metric that can be used to evaluate the performance of GCN in classifying positive and negative edges. The higher the auROC value is, the better the performance of the model will be in distinguishing positive and negative edges within the graphs. An auROC value of 0.5 and below corresponds to the worst performing model and a value of 1.0 corresponds to the best performing model.

The “*FullBatchLinkGenerator*” function was used to apply a binary operator to classify the relationship between sgRNA and target as positive and negative links, 1 and 0, respectively. A value of 1 denotes that the link exists between sgRNA and target DNA sequence. A value of 0 denotes that the link does not exist between sgRNA and target sequences.

The GCN model was created using StellarGraph. GCN layers are stacked with graph convolution and dropout layers. A total of 2 GCN layers with 16 units each were used with a rate of dropout for input of each layer set to 30%. The output of each GCN layer was activated using *ReLu* activation. The *Adam* algorithm was used to optimize loss function with the learning rate set to 0.01 to train the model. The output of the model was a binary classification of 1 and 0 (1 denoting the presence and 0 indicating the absence of links between sgRNA and target sequences).

The performance of the model was evaluated by learning node and link embeddings. For node embedding, StellarGraph provides an option to evaluate and compute the performance of GCN model based on random walks based node embedding. A biased random walks was generated from the off-target graph with fixed random walk parameters of *p* (“1/p” probability of returning to source node) and *q* (“1/q” probability of moving to a node away from source node) set to 1. The model learns about the sequences (nodes) co-occurring in short random walks represented closely in the embedding space. Sequence representations were obtained and a binary classifier was used to predict if a link should exist between any two sequences in a graph.

A logistic regression classifier was trained on the embeddings of positive and negative edges to predict a binary value indicating if a link between the edges should exist or not. StellarGraph provides the option to evaluate the performance of the model using different binary operators—Hadamard, average, L1 and L2. The model was trained end-to-end using binary cross entropy between link probabilities and true link labels for 10 epoch values and evaluated using the test set. Finally, the model was applied to the independent test graph and auROC value was calculated.

## 5. Results and Discussion

The AUC values under ROC curves (auROC) are calculated for different scenarios to validate the performance of the model. As the NOT sequences are randomly sampled, the auROC values tend to change for every run. Hence, the experiment was run multiple times and the average auROC value is computed and shown in Table 2.

**Figure 6:**
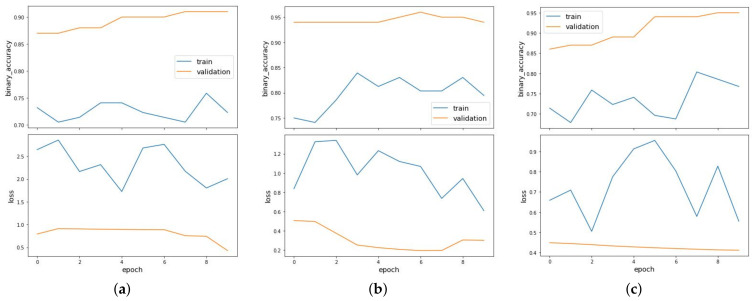
Binary accuracy and loss curves plotted for link prediction using occurrences of nucleotides with *k*-mer size of 1 as features for balanced (**a**), imbalanced_NOT (**b**) and imbalanced_OT (**c**) clusters.

**Figure 7:**
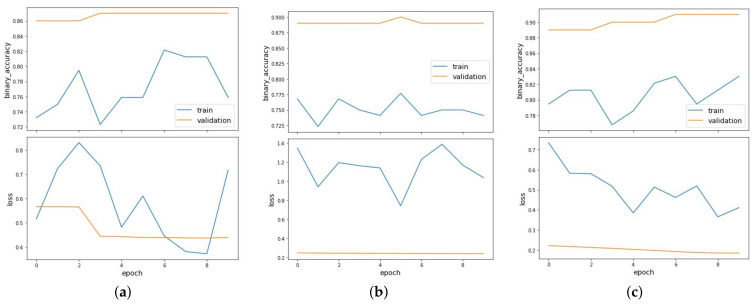
Binary accuracy and loss curves plotted for link prediction using occurrences of nucleotides with *k*-mer size of 2 as features for balanced (**a**), imbalanced_NOT (**b**) and imbalanced_OT (**c**) clusters.

**Figure 8:**
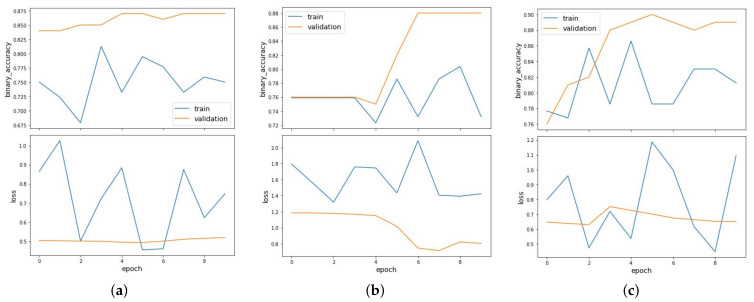
Binary accuracy and loss curves plotted for link prediction using occurrences of nucleotides with *k*-mer size of 3 as features for balanced (**a**), imbalanced_NOT (**b**) and imbalanced_OT (**c**) clusters.

It can be observed that the model performs well when the nucleotide occurrences extracted from sequences are given as features with *k* value as 1 in both the balanced and imbalanced scenarios. The GCN is able to achieve AUC value of 0.954 when the dataset is balanced. Figure 6 shows the binary accuracy and loss curves plotted for link prediction using occurrences of nucleotides with *k*-mer size of 1 as features for balanced (a), imbalanced_NOT (b) and imbalanced_OT (c) clusters. Under the imbalanced datasets, the model has an AUC value of 0.976 and 0.987, when the dataset is imbalanced towards OT and NOT sequences, respectively. Figure 7 shows the binary accuracy and loss curves plotted for link prediction using occurrences of nucleotides with *k*-mer size of 2 and Figure 8 with *k*-mer size of 3 as features for balanced (a), imbalanced_NOT (b) and imbalanced_OT (c) clusters. GCN performs very well in the imbalanced towards NOT scenario, where the number of negative samples are more than positive samples, which is similar to reality.

**Figure 9:**
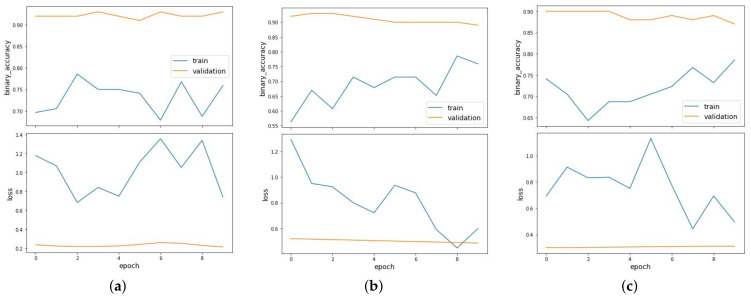
Binary accuracy and loss curves plotted for link prediction using position of nucleotides as features for balanced (**a**), imbalanced_NOT (**b**) and imbalanced_OT (**c**) clusters.

GCN also performs well when providing the position of the nucleotides in the sequences as features with auROC value of 0.925. From the results, it can also be observed that the performance of GCN reduces when we increase the *k*-mer sizes more than 1 as the auROC values were observed to less than 0.9 in some scenarios. Figure 9 shows the binary accuracy and loss curves plotted for link prediction using position of nucleotides as features for balanced (a), imbalanced_NOT (b) and imbalanced_OT (c) clusters. In all the scenarios, the model has achieved an auROC value above 0.8, which proves that the performance of GCN is excellent in predicting off-target efficacy of sgRNA in CRISPR/Cas9 gene editing.

We compared the performance of GCN model with previous deep learning models that were trained and evaluated on this off-target dataset in Table 3. We could see that our model performed better with high auROC values. We did not compare the performance of our model with *CNN_Std* model, as this model was evaluated on a different dataset.

In this research, a successful implementation of graph-based approach to predict off-target mutations in CRISPR/Cas9 system is achieved. The main findings are that:

1. A graph-based approach to predict off-target mutations in CRISPR/Cas9 gene editing, which is easy to implement and replicate by researchers, is possible. Link prediction is done to predict the presence of links between sgRNA and off-target inducing target DNA (OT) sequences.
2. GCN, a powerful graph neural network model used for performing representation learning of graphs, can predict off-target efficacy in CRISPR/Cas9 gene editing by performing link prediction on the off-target dataset.
3. Sequence-based features, such as position and occurrences of nucleotides in a sequence can increase the performance of GCN model in analysing graphs with high accuracy. GCN is used to validate the off-target efficacy of sgRNA in both these feature types. Performance of GCN is extremely good, when nucleotide occurrences with *k*-mer size of as 1 is given as features for the sequences in the network graph. Providing the position of nucleotide in a sequence as a feature for nodes in the graph is important in off-target prediction and GCN can predict with high auROC values.
4. The off-target dataset is heavily imbalanced and cluster data sampling is done to overcome this imbalance issue by randomly sampling the majority class, NOT sequences with respect to the count of OT sequences in each sgRNA cluster.
5. StellarGraph, a user friendly API, can be used to create network graphs and perform validation using a GNN model, if the off-target dataset is not highly imbalanced, as this API provides many in-built codes to enable researchers to automate the creation and normalization of adjacency and feature matrices without much manual effort.

## 6. Conclusions

In this approach, we introduced a graph-based approach to predict the off-target efficacy of sgRNA using the link prediction method by which the existence of links between sgRNA and target DNA sequences is predicted. We could see that the models were able to achieve high AUC values under ROC curves (auROC) when predicting off-targets. To our knowledge, this is the first time graph neural networks have been designed and implemented for off-target predictions. Unlike the previous deep learning models, this approach is easy to understand and replicate for off-target prediction research. Link prediction was performed using StellarGraph and made the computation process much easier. We conclude that graph convolutional networks can improve the predictive performance of sgRNA off-target activity.

## Figures and Tables

**Table 1:** Cluster data balancing of sequences in balanced and imbalanced clusters.

Cluster Data Samples	sgRNA	OT	NOT
Imbalanced_OT Clusters (OT > NOT)	29	626	304
Balanced Clusters (OT = NOT)	29	626	626
Imbalanced_NOT Clusters (OT < NOT)	29	626	1252

**Table 2:** AUC values under ROC curves (auROC) computed in link prediction.

Feature Types	Balanced Clusters	Imbalanced_NOT Clusters	Imbalanced_OT Clusters
Nucleotide Occurrence (*k* = 1)	0.954	0.987	0.976
Nucleotide Occurrence (*k* = 2)	0.889	0.914	0.931
Nucleotide Occurrence (*k* = 3)	0.888	0.888	0.893
Nucleotide Position	0.925	0.884	0.879

**Table 3:** Comparison of AUC values under ROC curves (auROC) computed by different models on the same off-target dataset.

Model	auROC Value
DeepCrispr	0.857
AttnToMismatch_CNN	0.970
CnnCrispr	0.984
GCN-CRISPR	0.987

## Data Availability

Data used in this study is included in the published articles “DeepCrispr” and “CnnCrispr”. The corresponding supplementary information files can be found below: DeepCrispr—https://doi.org/10.1186/s13059-018-1459-4, accessed on 14 May 2021. CnnCrispr—https://doi.org/10.1186/s12859-020-3395-z, accessed on 14 May 2021. The data can also be downloaded from *CnnCrispr* [44] and the file name is “*off-target data*”.
